# Content-specific coordination of listeners' to speakers' EEG during communication

**DOI:** 10.3389/fnhum.2012.00266

**Published:** 2012-10-01

**Authors:** Anna K. Kuhlen, Carsten Allefeld, John-Dylan Haynes

**Affiliations:** ^1^Bernstein Center for Computational Neuroscience, Charité – Universitätsmedizin BerlinBerlin, Germany; ^2^Berlin School of Mind and Brain, Humboldt-Universität zu BerlinBerlin, Germany; ^3^Berlin Center of Advanced Neuroimaging, Charité – Universitätsmedizin BerlinBerlin, Germany; ^4^Excellence Cluster NeuroCure, Charité – Universitätsmedizin BerlinBerlin, Germany; ^5^Department of Psychology, Humboldt-Universität zu BerlinBerlin, Germany

**Keywords:** communication, spoken language, interpersonal coordination, dual EEG, social interaction, situation model, language production, language comprehension

## Abstract

Cognitive neuroscience has recently begun to extend its focus from the isolated individual mind to two or more individuals coordinating with each other. In this study we uncover a coordination of neural activity between the ongoing electroencephalogram (EEG) of two people—a person speaking and a person listening. The EEG of one set of twelve participants (“speakers”) was recorded while they were narrating short stories. The EEG of another set of twelve participants (“listeners”) was recorded while watching audiovisual recordings of these stories. Specifically, listeners watched the superimposed videos of two speakers simultaneously and were instructed to attend either to one or the other speaker. This allowed us to isolate neural coordination due to processing the communicated content from the effects of sensory input. We find several neural signatures of communication: First, the EEG is more similar among listeners attending to the same speaker than among listeners attending to different speakers, indicating that listeners' EEG reflects content-specific information. Secondly, listeners' EEG activity correlates with the attended speakers' EEG, peaking at a time delay of about 12.5 s. This correlation takes place not only between homologous, but also between non-homologous brain areas in speakers and listeners. A semantic analysis of the stories suggests that listeners coordinate with speakers at the level of complex semantic representations, so-called “situation models”. With this study we link a coordination of neural activity between individuals directly to verbally communicated information.

## Introduction

Much of what we humans do, we do within a social context and in interaction with other human beings. In contrast, traditional approaches in cognitive psychology and cognitive neuroscience tend to focus on the isolated individual mind (for a similar view, see e.g., Sebanz et al., 2006; Hari and Kujala, 2009; Wilms et al., 2010; Kuhlen, 2011). Even when the topic of investigation is social, researchers often limit themselves to investigating how the *individual* mind processes social information (de Jaegher et al., 2010). In social interactions, however, two (or more) minds come together: Individuals coordinate and adapt to each other. To understand the underpinnings of this process of coordination it is therefore necessary to relate two individuals' cognitive and neural states to each other. The present study investigates a prototypical context in which individuals coordinate: spoken communication (Clark, 1996). Specifically, our study examines how neural activity, measured through recordings of the ongoing electroencephalogram (EEG) of two individuals, coordinates during communication. In order to increase experimental control we here restricted ourselves to unidirectional communication, where one individual is speaking and the other listening.

During face-to-face communication, conversational partners monitor and coordinate their current level of understanding in a collaborative process known as *grounding* (e.g., Clark and Brennan, 1991; Clark, 1996). Even when there is no possibility for mutually negotiating meaning, as during unidirectional communication, conversational partners closely coordinate their understanding. For example, when listening to a recorded monologue on a shared visual scene, listeners' gaze coordinates with the recorded speakers' gaze, indicating their degree of understanding (Richardson and Dale, 2005). Not only gaze, various aspects of linguistic and nonlinguistic behavior, such as lexical or syntactic expressions and gestures, coordinate during communication (for a recent review see Branigan et al., 2010). Underlying such behavioral coordination is presumably a coordination of shared mental representations that accumulate in the minds of the communicating individuals as the conversation unfolds (Clark and Brennan, 1991; Pickering and Garrod, 2004).

Recent functional neuroimaging studies have investigated a coordination of neural activity between unidirectionally communicating individuals. For example, Anders and colleagues (2011) were able to predict the brain activity of a person interpreting an affective facial display based on the brain activity of the person displaying it. Analyzing pairwise homologous brain areas, the authors identified a neural network that was activated both while producing and comprehending nonverbal messages. Along similar lines, Schippers and colleagues (2010) found a coordination of neural activity between one individual communicating through pantomimic gestures with another by applying between-brain Granger-causality. And finally, Stephens et al. (2010) compared the brain activity of an individual telling a story with the brain activity of individuals listening to this story. Here, a one-to-one correlation between voxels revealed that the brain activity of the listeners coordinated with the brain activity of the speaker.

These studies suggest that during an exchange of communicative messages, individuals coordinate by activating primarily homologous brain areas. This is in line with psycholinguistic theories that assume that processes involved in producing a communicative message draw upon similar representations as processes involved in comprehending the message (e.g., Mattingly and Liberman, 1988; Calvert et al., 1997; Liberman and Whalen, 2000; Pickering and Garrod, 2004; Galantucci et al., 2006). But a coordination of neural activity is not necessarily restricted to the activation of homologous brain areas. Following Bressler and Kelso (2001), coordination may be generally defined as a “functional ordering among interacting components” (p. 26), meaning that the state of one of the components places constraints upon the possible states of the others. Homologous activation patterns are therefore only one special case of interpersonal coordination. However, the methods of data analysis used by Stephens et al. (2010) and Anders et al. (2011) did not account for the possibility of coordination involving non-homologous areas, in part due to the high-dimensional structure of neuroimaging data. When analysis was not restricted to coordination between homologous brain areas only, non-homologous areas, as well have been reported to support inter-personal coordination (Schippers et al., 2010). It therefore remains to be systematically investigated whether interacting individuals coordinate predominantly homologous or non-homologous brain areas.

In the present study we adopt the experimental setting of unidirectional spoken communication, but use EEG to observe neural coordination between communicating individuals. Compared to fMRI studies EEG has the advantage of a high temporal resolution that allows investigating in detail the timing of interpersonal coordination. Furthermore, EEG has the advantage that it is comparatively unobtrusive and thus allows an investigation of communication under more natural circumstances. EEG has recently been used to investigate neural coordination between two people interacting. Social interaction has been approximated in various domains, for example, by observing individuals while they were playing a game of cards (Astolfi et al., 2010), tapping their fingers in synchrony (Tognoli et al., 2007), imitating each other's hand movements (Dumas et al., 2010), or playing guitar together (Lindenberger et al., 2009). While these studies were able to observe coordination in bidirectionally interactive settings, most of them focused on two individuals acting simultaneously or performing identical actions (but see Astolfi et al., 2010). But this makes the reported synchronicity of neural activity difficult to interpret: It could be due to a coordination between the individuals acting jointly, or simply due to them acting in parallel but in isolation from each other. The restriction to unidirectional communication allows us to design our experiment using an attentional manipulation, thereby enabling us to disentangle a similarity of neural activity due only to common sensory input or motor action from a coordination that is due to the processing of communicated content.

**Figure 1:**
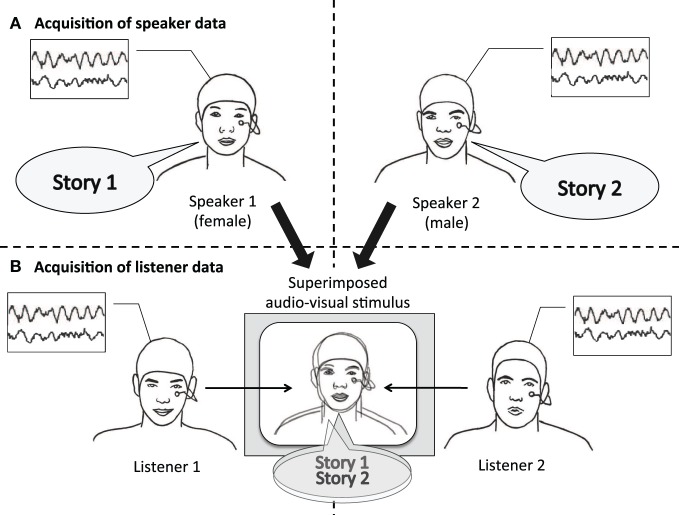
**Experimental design.** Speaker and listener data were acquired separately. **(A)** Speakers narrated short stories while video and EEG was recorded. **(B)** For each stimulus, video and audio of one male and one female speaker were superimposed. Audiovisual stimuli were presented to listeners with the instruction to attend either to the female speaker (listener 1), or to the male speaker (listener 2). Listeners' EEG was recorded while attending to one of the stories.

In our experiment, we first recorded a person telling a story (“speaker”) and later presented another person with an audiovisual recording of this story (“listener”). We then relate the EEG signal of the speaker to the EEG signal of the listener. To ascertain that an observed neural coordination is due to processing communicated content, audiovisual recordings of two speakers were superimposed and presented simultaneously, and listeners were instructed to attend either to one or to the other speaker (see Figure 1). Thus, sensory input was identical across all listeners; what varied between listeners was whom they attended to. This way we narrow down our explanation for a possible neural coordination to the processing of communicated content and limit alternative explanations based on low-level auditory effects. We hypothesize (1) that the EEG of listeners systematically depends on which speaker they attended to, reflecting activity specific to the communicated content. In addition, we hypothesize (2) that listeners' EEG is more strongly coordinated with the EEG of the attended speaker than with the EEG of the unattended speaker.

## Materials and methods

### Participants

Twelve participants (6 males, 6 females) were recruited as *speakers* through an advertisement in a local online classifieds site. Speakers self-identified as enjoying telling stories. Through the same classifieds site a different set of 12 participants (four males, eight females) were recruited as *listeners*. Listeners self-identified as enjoying listening to stories. Both speakers and listeners were native German speaking students, between 18 and 35 years old, and right-handed. All participants gave their written informed consent according to the declaration of Helsinki and received a compensation of 10 € per hour for their participation. Due to a recording error, the data of one listener were lost for one of the stories.

### Acquisition of speaker data

#### Story material

Each speaker told five stories in total. Four stories were randomly selected from a collection of 15 fairytales. These fairytales were taken from a book of “international fairytales,” collected from all over the world, so that the plots and characters were unfamiliar to participants. Speakers read, and then recounted them. For the fifth story speakers were asked to recount the plot of their favorite movie or book. This yielded a corpus of 60 stories (48 versions of assorted fairytales, 12 unique narrative summaries) from which the stimulus material for the listeners was selected. Additional data consisting of speakers giving spatial directions were collected as a pilot for future studies, but were not part of the current analysis. Story retellings lasted on average 3.77 min (*SD* = 1.38 min) and consisted on average of 611.5 words (*SD* = 216.74 words).

#### Procedure

Speakers were comfortably seated with their hands resting on a table in front of them to minimize movements. The video camera was located on the opposite side of the table. During the application of the EEG cap speakers had sufficient time to read and prepare the selected stories until they felt ready to later reproduce them in their own words. Speakers were given the task to make the stories interesting and fun for future listeners to listen to. To give speakers a minimal audience, the experimenter sat across of speakers, but speakers were instructed to direct their storytelling to the camera.

### Acquisition of listener data

#### Stories selected for stimuli

From the corpus of recorded stories, eight fairytales and eight narrative summaries were selected to be played back to the listeners. Each selected recording was paired with a story of similar duration, but different narrative content. Within each pair there were one male and one female speaker. These pairs of recordings were edited with Adobe Premiere Pro CS5.5 to superimpose the videos of the two speakers' faces and the soundtracks of their voices onto each other. Five independent raters adjusted the transparency of the recordings and the sound volume so that the two speakers appeared to be equally prominent.

Superimposed recordings (each hereafter referred to as a “stimulus”) were presented to two groups of listeners. One group was instructed to attend to one speaker, the other group to the other speaker. Within each group, half of the speakers that were attended to were female. In total each listener was presented with eight stimuli. To cue listeners which speaker to attend to, the first 5 s of each stimulus showed only that speaker, without superimposing the other one.

#### Procedure

After mounting the EEG cap, listeners were seated in front of a computer screen and initiated the playback of the stimuli upon notice by the experimenter. The order of the stimuli followed a balanced Latin Square design. Listeners were informed that they would be tested on details of the attended story following each stimulus presentation.

#### Behavioral assessment

After each story listeners were asked to answer seven multiple choice questions pertaining to details of the attended story (each with five possible answers). In addition, listeners were asked to indicate on a 7-point Likert scale how well they had been able to concentrate on the assigned speaker.

### Apparatus and setup

#### Recording and playback of video

Speakers' stories were recorded with a Canon Legria HD-Camcorder supported by a Sony ECM-MS 957 stereo microphone with 90° directionality. Recordings were played back to listeners on a MacBook Pro laptop with a 15″ screen supported by Creative D100 loudspeakers.

The conditions during the recording of the stories (room illumination, position of camcorder, microphone, and speaker's chair) and during the playback of the stimuli (sound volume, screen luminance, and position of computer and loudspeakers) were kept identical across all subjects.

#### EEG data acquisition

Electroencephalographic data were continuously recorded using a BrainAmp MR amplifier (Brain Products, Munich, Germany) at a sampling rate of 500 Hz, with analog filters at 250 Hz (anti-aliasing high-pass) and 0.1 Hz (detrending low-pass). EEG signals were recorded from 62 scalp locations positioned according to the International 10/20 System (American Electroencephalographic Society, 1994) using Ag/AgCl electrodes connected to the skin with abrasive electrolyte gel. Voltages were measured versus FCz, and re-referenced offline to the average reference (recovering the FCz channel). Impedances were kept below 5 kΩ. Eye movements were monitored via an EOG (electrooculogram) electrode fixed below the left eye. From each subject, we additionally recorded resting EEG, 1 min with eyes closed and eyes opened each.

### EEG preprocessing

#### Alignment of speaker and listener EEG

For a precise synchronization of the EEG recording and video recording and playback, the corresponding audio signal was fed into the EEG amplifier by converting an unused ECG (electrocardiogram) electrode. This resulted in a low-quality audio recording being included in the EEG data file (used for synchronization purpose only). The EEG of each subject was aligned with the video recording or with the presented stimulus, respectively, by computing the cross-correlation function between the “ECG”-audio and the down-sampled audio from the video recording. The correct alignment was estimated by the maximum of the absolute value of the cross-correlation. To correct for a possible imperfect separation of channels in the amplifier (“cross-talk”), the down-sampled audio was subsequently regressed out of all remaining EEG channels. Remaining low-amplitude audio components were identified and removed in the general artifact removal step (see below).

For each stimulus the set of EEG recordings from the two speakers and the twelve listeners was temporally aligned based on the previously synchronized corresponding audio signals. Because of non-overlapping segments due to different story lengths or slightly different start and stop times, recordings were trimmed to the overlapping time segment.

#### Artifact removal

Line noise artifact was suppressed by applying notch filters at 50 Hz and integer multiples. Further signal components of non-neural origin, most importantly electromyogenic artifacts (mostly due to speaking) and eye artifacts (blinking and eye movements) were removed using a procedure based on independent component analysis (ICA).

ICA aims to separate signal components of different origin, such that artifactual components can be identified and removed. For each subject and recording separately, we decomposed the 63-channel data set (including the EOG channel, and with appended eyes-closed and eyes-open recordings) into independent components using the DSS implementation (Särelä and Valpola, 2005) of FastICA with a tanh nonlinearity (Hyvärinen, 1999), as included in the FieldTrip toolbox (Oostenveld et al., 2011). Three independent raters then rated components regarding their degree of contamination on a 7-point scale (in increments of 0.5 from 1 = “pure neurogenic” to 4 = “pure artifact”). Ratings were based on the components' topography, time series, and power spectrum, following recommendations on component classification by McMenamin et al. (2010, 2011).

For the development and training of the rating scheme, the raters used 756 components from story recordings that did not enter the final data set. To assess reliability of raters' judgments, 504 components (one third of the speaker data that entered the final data set) were classified by each of the raters independently. Inter-rater reliability, evaluated by the intraclass-correlation coefficient (Shrout and Fleiss, 1979), was high, ICC = 0.88. Disagreements were discussed and the mean value of all three ratings was subsequently used. After this initial process of calibration, each rater then rated a portion of the remaining 9009 components from the listener data. During this phase inter-rater reliability was re-assessed at two more time points (each time on another 504 components from the speaker data), and remained high throughout the rating process (ICC = 0.86 and ICC = 0.88, respectively).

Components rated as purely or predominantly driven by artifacts (rating >3.0) were excluded from further analysis. Remaining components were projected back into the space of the original 63 channels, and the EOG channel was discarded.

### EEG analysis

All analyses were performed on the pre-processed EEG voltage data. Narration types (fairytales and narrative summaries) were collapsed in the main analysis. For assessing the reliability of our findings, we also performed and report analysis results separately for story types. In this section we summarize the main aspects of the EEG data analysis. For a complete description please refer to the Appendix, which also motivates our approach using a model of the speaker–listener coordination process.

#### Analysis of content-specific activity in listeners

First, we identify within the listeners' EEG the component that is specific to the content of the story they attended to. For this we extract from the signal (voltage as a function of time and channel) the component that is common among listeners attending to one story and different from listeners attending to the other story (in the same stimulus). This signal component accounts for a proportion of the total variance of the listeners' EEG, denoted *R*^2^_*L*_. This measure gives the size of the specific effect the story content has on the listeners' EEG. Results are averaged across stimuli.

#### Analysis of content-specific correlation between speakers and listeners

Secondly, we investigate whether there is a correlation between the listeners' EEG and the EEG of the speaker they attended to. The correlation is not computed for each single listener, but with respect to the content-specific component of the EEG common to all listeners attending to the same speaker. The listener analysis described above therefore serves as a preprocessing step for the speaker–listener analysis.

To account for the possibility that activity coordinated between speakers and listeners appears in homologous or non-homologous areas, we used canonical correlation analysis (Hotelling, 1936; Mardia et al., 1982). This approach includes the possibility for signal components that are common between speaker and listeners to appear in arbitrary combinations of EEG channels. The result is a measure of *set correlation* (Cohen, 1982), a generalization of the Pearson correlation between two signals to the case where each “signal” is a multivariate data set. The measure quantifies the proportion of generalized variance, *R*^2^_*SL*_, shared between the two multivariate data sets, attended speaker EEG and listener EEG. Results are averaged across stimuli and speakers.

In order to investigate whether the observed set correlation is at least partially due to activity homologous in both speaker and listeners, we also computed a variant of our measure. This measure, *R*^2^_1:1_, is computed in the same way as the set correlation *R*^2^_SL_, with the modification that it is based on channelwise one-to-one correlations only (e.g., between the Cz electrode in the speaker and the Cz electrode in the listener).

#### Time lags

Listeners' cognitive and neural processes may lag behind those of the speaker (e.g., listeners needing time to process the input), or may precede (e.g., listeners anticipating what comes next). For this reason, the same correlation analysis was performed at different time lags, between +20 s (listener follows) and −2 s (listener anticipates) in steps of 0.5 s.

#### Topographies and frequencies

To obtain topographic information characterizing the content-specific activity in the listeners, analyses were also performed on single channels. For the coordination between speakers and listeners, the canonical correlation analysis itself provides topographic information in the form of a series of canonical modes (linear combinations). As an alternative, we also computed the measure of set correlation between seven subsets of channels of nine electrodes each (regions of interest). Frequency profiles of the effects were computed by combining the variance decomposition underlying all types of analyses with a variance decomposition by spectral analysis.

#### Relation between listener analysis and speaker–listener analysis

We performed two main analyses, aimed at content-specific activity in listeners and content-specific correlation between speakers and listeners. These two analyses are related to each other, insofar as every signal component contributing to a content-specific correlation must also have a content-specific effect on the listeners' EEG alone. However, the same does not hold in the other direction: There may be content-specific signal components in the listeners' EEG that do not have a counterpart in the speakers' EEG and consequently do not contribute to the speaker–listener correlation. The listener analysis therefore constrains the speaker–listener analysis, but does not determine it. This is especially important with respect to the specific frequency bands or scalp regions involved.

#### Statistical significance and bias correction

Hypothesis tests are based on a common permutation framework: All analyses are performed not only on the real data but also on permuted data, in which listeners are exchanged between the two groups with different attentive focus while keeping the group sizes constant. This procedure realizes the common null hypothesis that it does not make a difference which speaker a listener attends to. The resulting permutation distribution of values of *R*^2^_L_, *R*^2^_SL_, and *R*^2^_1:1_ is then used to determine the *p*-value of the observed effect. The permutation approach is also used to obtain *p*-values corrected for multiple testing.

Additionally, we use the permutation distribution to compute the estimation bias of *R*^2^ and correct for it (see “Appendix”). We report the bias-corrected measures, denoted as Δ*R*^2^_L_, Δ*R*^2^_SL_, and Δ*R*^2^_1:1_, which quantify the *increase* in the amount of explained or shared variance relative to the null hypothesis.

## Results

### Listeners were able to attend to one speaker

Listeners answered correctly on average 66% (*SD* = 25.7%) of the multiple-choice questions on details in the stories (chance level: 14.28%). This indicates that listeners were able to follow the speaker they were instructed to attend to, although they may not always have understood every detail. Listeners subjectively rated their ability to concentrate on the narration on a 7-point scale (7 = “bad concentration”) with a mean score of 3.39 (*SD* = 1.68).

### The EEG of listeners reflects content-specific activity

The two groups of listeners with different attentive focus show a systematic difference in their EEG: The multivariate analysis of variance results in a significant effect (*p* = 0.00216) of size Δ*R*^2^_*L*_ = 0.0336. This means that about 3% of the total variance of the listeners' EEG can be explained by taking into account which speaker they attended to, a considerable effect in view of the amount of endogenous background activity taking place in the human brain beyond task-related processes (compare Niedermeyer and Lopes da Silva, 2005; chapters 31 and 9).

**Figure 2:**
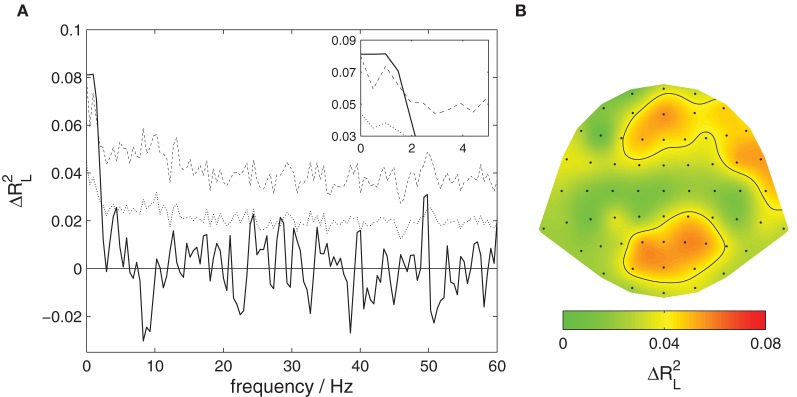
**Content-specific activity in listeners.** The proportion of variance of listeners' EEG explained by the attentive focus, Δ*R*^2^_L_, is decomposed across frequencies and EEG channels. **(A)** Contributions from different frequency components. The solid black line shows the observed proportion of explained variance, the dotted line the threshold for significance at a level of 0.05 uncorrected, the dashed line corrected for multiple comparisons at different frequencies. Significant contributions are found for the slowest signal components, frequencies below 3 Hz. This section is magnified in the upper right corner. **(B)** Contributions from different EEG channels. The scalp surface is shown in a top view so that right and left of the subject appear right and left in the plot. The ratio of explained variance is color-coded, the black contour delineates areas where the local effect is significant at a level of 0.05 corrected for multiple comparisons. Significant contributions are found at medial and right frontal as well as occipito-parietal locations.

Figure 2A shows a decomposition of this global effect into contributions from different frequency bands. Significant contributions (*p* < 0.05 corrected) are observed for very slow components of the listeners' EEG with frequencies below 3 Hz.

Figure 2B shows a decomposition into contributions from different channels. A significant effect (*p* < 0.05 corrected) is found over medial frontal, right frontal, as well as occipito-parietal scalp areas.

#### Reliability

Separate analyses for fairytales and narrative summaries yield significant effects (*p* = 0.0216 each) of sizes Δ*R*^2^_*L*_ = 0.0411 and 0.0260, respectively.

### Listeners' EEG coordinates with the EEG of the attended speaker

**Figure 3:**
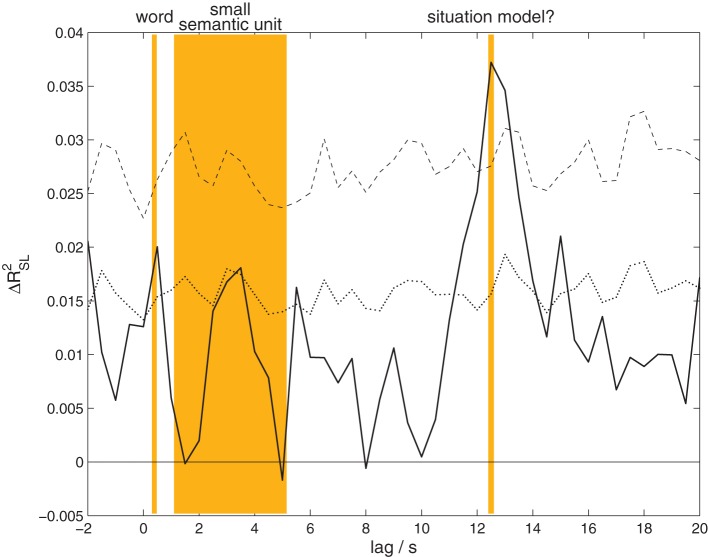
**Content-specific correlation between speakers and listeners.** The proportion of generalized variance shared between speakers' and listeners' EEG, Δ*R*^2^_SL_, at different time lags. A positive lag means that the analysis combines a later time point in the listeners' EEG with an earlier time point in the speakers' EEG. The solid black line shows the observed proportion of explained variance, the black dotted line the threshold for significance at a level of 0.05 uncorrected, the dashed black line corrected for multiple comparisons. A significant amount of shared variance is found at lags from 12 to 13.5 s, peaking at 12.5 s. The orange background indicates the typical lengths of words and small semantic units occurring in our experiment, as well as a possible interpretation for the observed correlation effect.

Listeners' EEG was more strongly correlated with the EEG of the attended speaker than the unattended speaker. The results of the canonical correlation analysis between the EEG of speakers and listeners at time lags from −2 to 20 s are shown in Figure 3. The analysis reveals a significant effect (*p* = 0.00108, corrected for multiple comparisons) peaking at a lag of 12.5 s (listeners following) with a maximum effect size of Δ*R*^2^_SL_ = 0.0372. That is, at this lag a proportion of almost 4% of the generalized variance of speakers' EEG is shared with the listeners' EEG. As detailed in the methods section, the effect size measure and statistics reported here are based on a comparison with the distribution obtained from permuted data, where listeners are exchanged between the two groups. Therefore, our results indicate that the correlation of the EEG with the attended speaker is larger than with the unattended speaker.

In order to have a basis for the interpretation of the observed time lag between speakers and listeners, we determined the average length of words and small semantic units in the stories. For this purpose all stories were transcribed, and the total duration of the story was divided by the number of words. Average word lengths ranged from 306 to 478 ms (5–95% quantiles across stimuli), mean 368 ms. In a second step, two independent raters segmented the transcripts into small semantic units. One small semantic unit was defined as a proposition or a set of propositions that advanced the plot of the story (e.g., “two brothers went into the woods”; for a similar analysis see Kuhlen and Brennan, 2010). Raters agreed on 92.48% of their segmentation decisions. According to this segmentation, one small semantic unit consisted of 3–14 words (5–95% quantiles for all stimuli aggregated), mean 7.62 words, corresponding to durations from 1.1 to 5.15 s. In the context of our measure of interpersonal coordination in the EEG, this suggests that the observed time lag corresponds to larger units in the story, consisting of an average length of 34 words or 4.5 smaller semantic units.

The global correlation effect between speakers and listeners at the lag 12.5 s was decomposed into contributions from different frequency bands. No statistically significant effect emerged in any specific set of frequencies. This indicates that the speaker–listener correlation is due to shared signal components spread over a broad range of frequency components.

The degree of coordination in the EEG of speakers and listeners did not correlate with listeners' performance in the multiple choice questionnaire testing details of the narrations.

#### Reliability

**Figure 4:**
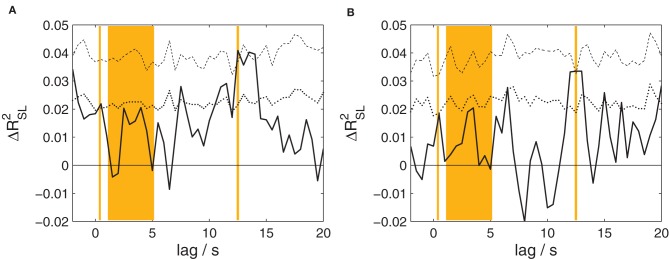
**The proportion of shared variance between speakers' and listeners' EEG, separately for the two types of stories.** Compare Figure 3 for details. **(A)** Results for fairytales. **(B)** Results for narrative summaries. For both types of stories, a statistically significant amount of shared variance is found at a lag of 12.5 s.

The canonical correlation analysis between the EEG of speakers and listeners at time lags from −2 to 20 s was also performed separately for fairytales and narrative summaries; the results are shown in Figure 4. Both analyses reveal a significant effect (*p* = 0.0162 and 0.040, respectively; corrected for multiple comparisons) peaking at a lag of 12.5 s in each case. Associated maximum effect sizes are Δ*R*^2^_SL_ = 0.0409 and 0.0335, for fairytales and narrative summaries, respectively.

### Coordination is not restricted to homologous brain areas in speakers and listeners

**Figure 5:**
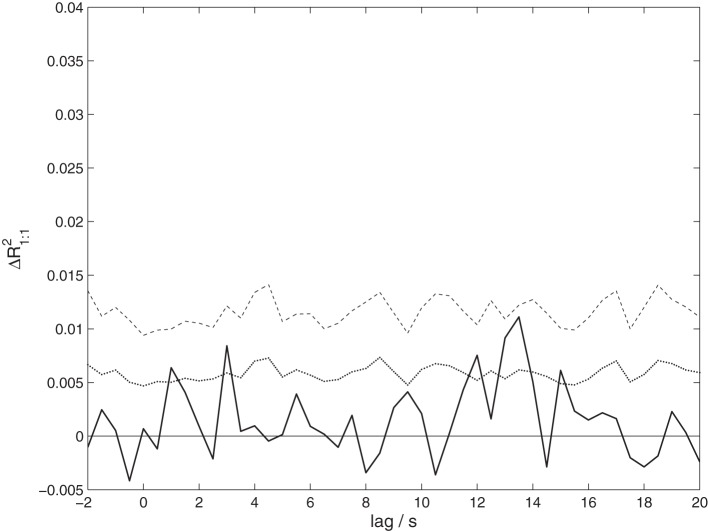
**The proportion of shared variance between speakers' and listeners' EEG, taking only correlations between corresponding EEG channels into account, Δ*R*^2^_1:1_.** Compare Figure 3 for details.

To test whether the correlation observed between speakers and listeners is due to activity in homologous brain areas, we performed a variant of the analysis taking only one-to-one correlations into account. The results shown in Figure 5 reveal only a non-significant trend (*p* = 0.0984 corrected) of maximal size Δ*R*^2^_1:1_ = 0.0111 at a lag of 13.5 s. Although this result is consistent with the previous analysis, it shows a much weaker effect. This suggests that the observed coordination does *not primarily arise from a co-activation of homologous brain areas*.

The statistical assessment of the canonical variates (see Mardia et al., 1982) at the lag of maximum Δ*R*^2^_SL_, 12.5 s, indicates that the first 17 variates contribute significantly at a level of 0.05. Associated canonical correlations decrease only very slowly (from *r*^2^_1_ = 0.0154). This indicates that the variance shared between speakers and listeners is due to a multi-dimensional signal component, which can not be characterized by one or a small number of scalp topographies.

**Figure 6:**
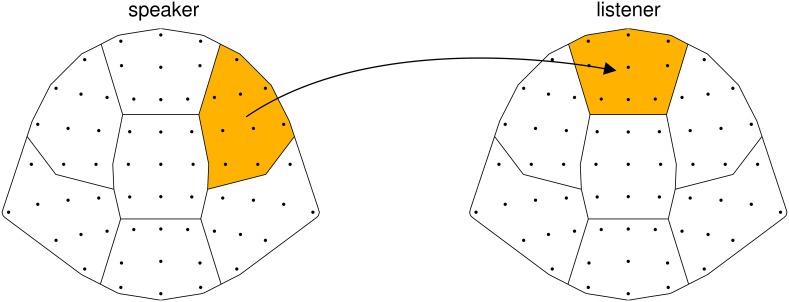
**Details of the correlation between speakers and listeners at lag 12.5 s, contributions from different scalp areas.** The proportion of generalized variance shared between speakers and listeners, Δ*R*^2^_SL_, computed between seven subsets of EEG channels (regions of interest). The black arrow indicates the significant correlation (at a level of 0.05 corrected) found between the right frontal area in speakers and the medial frontal area in listeners.

As an alternative, we assessed the spatial structure of correlations between speakers' and listeners' EEG using seven regions of interest (ROIs, see “Materials and Methods”), and computed Δ*R*^2^_SL_ for each of the resulting 49 pairs of ROIs separately. At a level of 0.05 corrected for multiple comparisons, only the combination of the right frontal ROI in speakers with the medial frontal ROI in listeners reached significance (Figure 6). Supporting the result of the one-to-one analysis, this suggests that the observed correlation is not mainly due to activity in homologous brain areas in speakers and listeners.

## Discussion

In social interactions, individuals coordinate not only their behavior but also their mental states. In this study, we identify a coordination of neural activity between the EEG of an individual telling a story (“speaker”) and the EEG of another individual listening to this story (“listener”). Our experimental design and analysis approach allowed us to link a coordination of electrophysiological activity between speakers and listeners to the processing of communicated content. Furthermore, the low-dimensional representation of brain activity given by EEG enabled us to use an approach to data analysis that can account for a coordination of not only homologous, but also non-homologous brain areas. And finally, the temporal resolution of EEG gave us important insights on the time scale at which speakers and listeners coordinate, namely that this coordination is based on slow processes and takes place at a time delay in listeners relative to speakers. In the following we will discuss our findings in relation to these aspects.

### Neural coordination reflects content-specific activity

Our experiment teases apart neural activity related to processing perceptual input from neural activity specific to the content of the story. We achieved this in two ways: Firstly, listeners' EEG recorded while attending to one story was compared to the EEG of listeners who had the same perceptual input but who attended to another story. We were able to show that the EEG is more similar among listeners attending to the same story. Secondly, the neural coordination we identified between speakers and listeners pertains to that component of the listeners' EEG that is specific to the content of the story. Our data show that listeners coordinated more strongly with the speaker they attended to than with the speaker they did not attend to. These findings strongly suggest that the observed neural coordination is indeed based on the processing of communicated information. In this respect, our work goes beyond previous studies that have used EEG to investigate social interactions, but did not link a similarity in neural activation directly to coordination processes during communication (Tognoli et al., 2007; Lindenberger et al., 2009; Astolfi et al., 2010; Dumas et al., 2010).

### Coordination between non-homologous brain areas

Our findings support previous neuroimaging studies that found neural coordination between two communicating individuals (e.g., Schippers et al., 2010; Stephens et al., 2010; Anders et al., 2011). In contrast to Anders et al. (2011) and Stephens et al. (2010), the coordination we found using EEG does not appear to be based primarily on the activation of homologous brain areas in speakers and listeners. This discrepancy may simply be due to the fact that these two studies exclusively looked at coordination between homologous areas. In contrast, when analysis was not restricted in this way, an additional involvement of non-homologous areas emerged (Schippers et al., 2010). Likewise, our analysis approach takes into account neural coordination between the multivariate speaker and listener data sets as a whole. This way our analysis was able to detect a coordination based on activation of non-homologous brain areas. In comparison, when restricting the analysis to one-to-one correlations between corresponding EEG channels, we found only a trend for speakers and listeners to coordinate. This is in line with other EEG studies that have investigated different types of social interaction and found coordination between non-homologous brain areas (e.g., while imitating gestures, Dumas et al., 2010; or playing cards, Astolfi et al., 2010).

The ROI-based identification of correlated scalp areas also suggests that listeners and speakers activate similar, but not identical areas: Speakers and listeners were similar in that both appeared to activate frontal scalp locations, suggesting a general involvement of higher cognitive functions (e.g., Frith and Dolan, 1996). But while the neural coordination on the speakers' side is based mainly upon activity picked up from right frontal electrodes, the neural coordination on the listeners' side is observed in medial frontal electrodes. This could indicate an additional involvement on the speakers' side of brain areas associated with retrieving information from memory (Shallice et al., 1994). On the listeners' side, the topography suggests an involvement of areas associated with social inference making and processes involved in observing the actions of others, such as the medial prefrontal cortex (e.g., Amodio and Frith, 2006). Due to the low spatial resolution of EEG, any such interpretation of our findings with respect to underlying brain areas is of course to be taken with caution. This is especially the case since topographies could be distorted due to the artifact-removal procedure, which may have attenuated components of the EEG that could not be separated from artifacts.

### Slow and delayed coordination between speakers and listeners

The identified inter-individual neural coordination appears to predominantly reflect slow processes that are characterized by large time scales. This is suggested on the one hand by the low-frequency components strongly contributing to the content-specific similarity between listeners attending to the same story. While effects in this frequency band are uncommon in cognitive studies, they are less surprising when considering that our stimulus presentations are very long. In the more common type of EEG studies, which use an event-related approach, the shorter and more frequent stimulus presentations may interrupt and thereby attenuate processes operating on larger time scales. A second and more striking indication that slow processes predominate the speaker–listener coordination observed in our experiment is the rather long delay at which listeners' neural activity reflects speakers' neural activity.

A detailed analysis of the narrative structure of our stories indicates that this delay corresponds to a time span in which speakers relate larger units of semantic information. These units could be interpreted as complex multidimensional representations of what is being discussed, so-called “situation models” (van Dijk and Kintsch, 1983; Zwaan and Radvansky, 1998; also: “mental model,” Johnson-Laird, 1983), which can require an integration of information from multiple sentences (e.g., who is doing what and where). The rationale for this correspondence would be that the more complex the representation that is being conveyed, and, correspondingly, the longer its verbal expression, the longer the delay at which a complete coordination of these representations is achieved. This interpretation is in line with recent cognitive accounts of interactive dialog, which propose that successful communication relies on an alignment of situation models between speakers and listeners (Pickering and Garrod, 2004).

Other studies investigating neural coordination between communicating individuals have also reported comparably long delays of up to 8 sec between the brain activity of the speaker and corresponding activity of the listener (Schippers et al., 2010; Stephens et al., 2010; Anders et al., 2011). Presumably this delay results from the time difference between the communicative message being planned and produced, relative to when it becomes comprehended. While these findings are based on fMRI data, which are known for comparatively poor temporal resolution, our considerably more time-sensitive EEG data with precisely synchronized recordings confirms the long delay at which listeners coordinate with speakers. We speculate that the time scale at which speakers and listeners coordinate may be further modulated by how quickly listeners can build up situation models. In our case, listeners may have been slowed down because the majority of our stories were unknown and possibly alien to them (half of the stories were international fairytales with unfamiliar plots and characters), which could have placed further demands on the listeners' capacity to understand what was going on (see e.g., Fincher-Kiefer et al., 1988). Future studies may investigate in detail which factors modulate the delay at which listeners coordinate with speakers.

Our results do not imply that speaker–listener coordination pertains *exclusively* to slow processes. According to hierarchical models of language processing (Kiebel et al., 2008; Pickering and Garrod, 2004), any coordination on higher levels associated with larger time scales rests on coordination at lower levels associated with shorter time scales. An analog view has been put forward with respect to the processing of complex visually related stories (Hasson et al., 2008). Though our experimental design and analysis method appear more sensitive to larger time scales (i.e., a coordination of situation models), the course of speaker–listener correlation across different time lags shows a tendency to coordination also at smaller time scales, including those corresponding to the typical length of words and smaller semantic units. These effects may not have reached significance because local word-by-word understanding may have been impaired due to an interference of the second, unattended story. Nonetheless listeners would be able to coordinate with speakers on the more global level of situation models by inferring missing details from the context.

Consistent with the point that coordination takes place at many different time scales is our finding that contributions to the speaker–listener correlation are spread out over a broad range of frequencies. Contrary to a common interpretation of EEG frequency bands (see Buzsáki, 2006), this suggests that it is not specifically oscillatory signal components that contribute to the correlation. Rather, we conjecture that our findings are based on changes in the recurrence of particular instantaneous scalp voltage distributions, which have recently been shown to exhibit fluctuations characterized by a large range of different time scales (van de Ville et al., 2010). Such scalp topographies reflect the current state of electrical activation of the brain (compare Appendix), which can be interpreted as reflecting the processing of particular sets of representations (Michel et al., 2009). Just as we interpreted time lags in the speaker–listener correlation to correspond to units of different *lengths* within the communicated message, frequencies would correspond to the *rate* at which linguistic units follow each other in the process of producing and comprehending speech.

An apparent discrepancy arises from the fact that while contributions to content-specific activity in listeners come predominantly from low-frequency components, the speaker–listener correlation cannot be pinpointed to this frequency band. However, as discussed above, constraints between the two types of analyses exist only in one direction: Activity in listeners that underlies the speaker–listener correlation also has to show up as content-specific activity in listeners alone—but not vice versa. Accordingly, the broad-band nature of speaker–listener correlation tells us that content-specific activity in listeners is not confined to low frequencies. This interpretation is supported by the observation (not reported) that the listener analysis applied to filtered data, where only frequency components below 4 Hz are retained, shows considerably weaker effects. A parallel argument explains why the topographies resulting from the listener-only analysis differ from the topographies resulting from the speaker–listener analysis: the area involved in speaker–listener correlation on the listeners' side (medial frontal) is only one of those showing content-specific activity.

### Recording EEG during spoken communication

Studies recording EEG during speech production are still rather uncommon (for a recent review of this emerging research area see Ganushchak et al., 2011). Our study demonstrates that it is possible to extract meaningful results from EEG data that are recorded while participants are speaking. In an extensive preprocessing step, ICA components of our EEG recordings were carefully inspected and removed if they showed a large degree of contamination by signals of non-neural origin. Despite this thorough cleaning of the EEG we cannot, of course, exclude the possibility that some artifactual activity remained in our data. But artifactual activity cannot account for the fact that we find a reliable correlation between speakers and listeners depending on whom listeners paid attention to. Moreover, the associated topographies make a dominant involvement of artifacts unlikely. We believe that our procedure for dealing with artifacts due to speech production (based on McMenamin et al., 2010, 2011) is a promising approach for future EEG studies investigating spoken communication.

In the present study listeners had no means of influencing the actions of their conversational partners. The neural coordination we report therefore relies entirely on listeners adapting to speakers in a unidirectional fashion. A typical communicative situation is, of course, far from being a unidirectional transfer of information between a “sender” and a “receiver”. Listeners actively contribute and shape speakers' behavior (see e.g., Bavelas et al., 2000; Kuhlen and Brennan, 2010). For the sake of experimental control we deliberately simplified what it means to be communicating. Our current experiment is therefore only a first step towards a more complete understanding of the neural processes underlying communication. With a neurophysiological marker for unidirectional communication established, future studies will need to investigate how these neural processes are retained or modified in settings with reciprocal interaction. Despite these limitations, we believe that with this study we advance existing research protocols in the neurosciences towards investigating real-life interactions while retaining a degree of experimental control that could not be achieved “in the wild”.

### Conflict of interest statement

The authors declare that the research was conducted in the absence of any commercial or financial relationships that could be construed as a potential conflict of interest.

## Appendix

### Coordination model and analysis

#### Speaker and listener model state sequences

For one stimulus we consider the two speakers (1, 2), as well as all listeners presented with that stimulus, half of which attended to speaker 1 and the other half to speaker 2. We are interested in how the neurophysiological states of speakers and listeners are related to each other during storytelling and -listening. These states are functions of time *t*, changing throughout the narration; in other words, we are modeling sequences of states.

A speaker's state sequence (ST) is composed of the following subsystem states:

- a part determining (or rather, realizing) the process of narrating (SD) and
- a part unrelated to narrating (SU).

A listener's state sequence (LT) is composed of

- a part determined by general properties of the stimulus (LDG),
- a part determined by properties of the stimulus the listener specifically attends to, i.e., the narration of one of the speakers (LDS), and
- a part unrelated to the stimulus (LU).

Our hypothesis is that via the process of storytelling and -listening the listener coordinates with the speaker. This coordination is realized in such a way that SD determines LDS, mediated by the stimulus and modulated by attention.

Observations of states can be constructed in different ways from measured data. In our analyses, we treat the pre-processed multivariate EEG voltage signals as direct yet presumably incomplete representations of the speakers' and listeners' states, ST and LT. They have the form of vectors (indexed by the EEG channel *c*), which gives state sequences the form of data matrices (indexed by *t* and *c*).

Moreover, we make the assumption that within this state space the combination of the subsystem states detailed above into the whole observed state appears as a linear superposition, motivated by the linear superposition of electric fields from different neuronal generators in EEG (see Nunez and Srinivasan, 2005). After preprocessing, data matrices are additionally standardized to mean 0 and standard deviation 1 across time points and channels, to compensate for possibly different EEG signal amplitudes and baselines in different subjects.

#### Listener model and analysis

From the definition of the subsystem states follows:

- LDG is identical for all listeners presented with the same stimulus, but different for different stimuli.
- LDS is identical for all listeners presented with the same stimulus and attending to the same speaker, but different for different stimuli as well as for listeners attending to different speakers (in the following: attentive focus).
- LU is different for all listeners, stimuli, and attentive foci, and considered as different realizations of the same multivariate stochastic process with zero mean.

Under these assumptions the listener state model for one stimulus obtains the form

$${\text{LT}}_{ij}=\text{LDG}+{\text{LDS}}_{i}+{\text{LU}}_{ij},$$

where *i* = 1, 2 indexes attentive foci within a stimulus and *j* = 1 … 6 listeners within the group with the same attentive focus. This model fits into the multivariate analysis of variance (MANOVA) version of the multivariate general linear model (GLMM) framework (Mardia et al., 1982). The model has a constant regressor with associated gain LDG, two regressors indicating attentive focus with gains given by LDS_*i*_, and an error term LU_*ij*_. All the terms are time × channel matrices, i.e., each combination of time point and channel defines one dependent variable, and data from different listeners are treated as samples.

Based on this model we calculate a multivariate statistic to test whether the contribution made by LDS is different from zero, i.e., whether there is an effect of the listener attending to a specific speaker. Since the resulting error covariance matrix is very large and there are only a few samples, the estimate needs to be regularized (see Hastie et al., 2008). We choose complete regularization, meaning that we assume mutually uncorrelated errors with equal variance (elements of LU). With this, the test statistic *Lawley–Hotelling Trace* can be written as

$$T=\frac{n}{4(n-2)}\frac{\sum_{tc}{\left({\text{LDS}}_{2}\left(t,\;c\right)-{\text{LDS}}_{1}\left(t,\;c\right)\right)}^{2}}{{\bar{\frac{1}{n-2}\sum_{ij}{\text{LU}}_{ij}{\left(t,\;c\right)}^{2}}}^{tc}},$$

where *n* = 12 denotes the number of listeners and the bar denotes the average across time points and channels. This statistic is up to a factor identical to the squared Euclidean distance between the two group centroids of the data matrices (numerator), set into relation to the pooled error variance (denominator). It can be converted into a measure of effect size,

$$R_{L}^{2}=\frac{T}{1+T}$$

the proportion of explained variance within listeners.

The total observed effect can be separated into contributions from different EEG channels by repeating the analysis including only the respective channel, calculating the topography of the effect size. It can also be separated into contributions from different frequencies by combining the variance decomposition of the MANOVA with a variance decomposition by spectral analysis, for which we used Welch's (1967) modified periodogram method with a window length of 2 s and a Hamming taper.

#### Speaker model and speaker–listener analysis

Formally, the part of the speaker's state sequence realizing the narrating, SD, would be defined by being identical for all speakers telling the same story, and LDS would be a function of SD. Likewise, the parts of speakers' state sequence unrelated to the narrating, the different SUs, would be different realizations of the same stochastic process.

Since in our experiment each story is narrated by one speaker only, we have just this one observation and can therefore not distinguish SD from SU. Moreover, through electrophysiological data we only observe aspects of speakers' and listeners' states. Aspects of LDS that are not reflected in the data make the corresponding parts of SD appear irrelevant and therefore to be a part of SU, and vice versa. The only statement we can therefore make is that there is a probabilistic relationship (a correlation) between ST and LDS.

We assume that in the EEG space the states in the LDS sequence depend in a linear way on the states in the SD sequence. This may seem at odds with the fact that the dynamics between speakers and listeners has to be nonlinear in order to realize a process of coordination (see Bressler and Kelso, 2001). However, even when a dynamics is nonlinear, the relation between dynamical variables established by it can in many cases be described by linear equations, at least as a useful first order approximation (see Wackermann, 1999). A possible alternative nonlinear implementation of our basic model of speaker–listener coordination would therefore just be an extension of the analysis described here.

If we allow for linear dependencies between ST and LDS to appear in arbitrary combinations of speaker and listener EEG channels, the model obtains the form

$${\text{LDS}}_{i}{\text{= ST}}_{i}\text{B +}\Xi_{i},$$

where *i* indexes attentive foci and corresponding speakers, B characterizes the linear function, and Ξ stands summarily for irregular signal components (“noise”) on both sides (including SU and LU). This model fits into the canonical correlation analysis (CA) version of the GLMM (Mardia et al., 1982), where LDS, ST, and Ξ are time × channel matrices and B is a channel × channel matrix. That means each EEG channel defines one independent and one dependent variable (on the speaker's and listeners' side, respectively), and data at different time points are treated as samples.

Based on this model we calculate a multivariate statistic to test whether there is a linear relationship between the speaker's EEG and that of the listeners. The test statistic *Wilks' Lambda* can be written

$$\Lambda_{i}=\frac{\left|\text{cov}\left({\text{ST}}_{i},{\text{ LDS}}_{i}\right)\right|}{\left|\text{cov}\left({\text{ST}}_{i}\right)\right|\left|\text{cov}\left({\text{LDS}}_{i}\right)\right|}$$

where |·| denotes the determinant and cov the covariance matrices within and between the channels of ST_*i*_ and LDS_*i*_, respectively. For each stimulus there are two such measures, one for each of the two speakers and the corresponding group of listeners. This test statistic can be converted into a measure of effect size,

$$R_{\text{SL}i}^{2}=1-\Lambda_{i},$$

the *set correlation*, which can be interpreted as the proportion of variance shared between ST_*i*_ and LDS_*i*_, if the concept of variance is generalized to the case of multivariate data sets (Cohen, 1982).

Since the coordination of listeners to speakers may not be instantaneous but may occur at an unknown delay, *R*^2^_SL*i*_ is computed at different lags by shifting one of the data matrices along the time axis and trimming the data to the overlapping time range. Parallel to the listener analysis, this measure can be separated into contributions from different frequencies by replacing the underlying covariance matrices by coherence matrices, estimated using Welch's modified periodogram method (see above).

Beyond a global measure of set correlation, CA results in a decomposition of the two data sets into a series of linear combinations that achieve maximal mutual correlation, the canonical components and associated canonical correlations. These linear combinations can be used to determine how strongly the respective component is present in the channels on the listeners' and speaker's side, providing effect size topographies for the CA.

As stated before, CA allows for correlations in arbitrary combinations of EEG channels, i.e., it is sensitive to both homologous and non-homologous processes. To investigate whether the observed set correlation is predominantly due to homologous activity, we also computed a variant of speaker–listener set correlation, *R*^2^_1:1_. It is computed in the same way as *R*^2^_SL_, with the modification that the non-diagonal part of cov(ST_*i*_,LDS_*i*_) is set to zero.

#### Statistical significance and effect size

Both of the investigated effects, the effect of the narration being attended to on the listeners' EEG and the relation between speaker's and listeners' EEG, are quantified using a measure of proportion of explained variance. Though *R*^2^_L_ was not introduced as such, it too can be interpreted as a set correlation, in this case between the MANOVA regressors and the listeners' EEG. We therefore have a unified form of effect size for the two different types of effects investigated.

Because our computations make use of regularization or use samples with serial correlation (time series), existing analytic approximations to the sampling distribution of *R*^2^ cannot be applied. To precisely assess statistical significance, we use a permutation approach (Good, 2005): All analyses are performed not only on the original data, but also on data where listeners are exchanged between the two groups who attend to different speakers. This procedure realizes the null hypotheses (1) that there are no systematic differences between the EEG of listeners attending to different narrations and (2) that there is no difference in the proportion of variance shared between a listener and the attended vs the unattended speaker, respectively. The *p*-value associated with a given set correlation can then be computed by determining its rank in the distribution of permutation values, which simulates the null distribution. There are 924 possible permutations for the distribution of 12 listeners into two equal-sized groups. For the assessment of the group difference in the listener analysis it is not important which label is attached to each group, and therefore only half of the permutations (462) are relevant.

Considered as an estimator of the population set correlation, *R*^2^ is severely positively biased. To obtain a more realistic estimate of the effect size we subtract the mean of the permutation distribution from log(1 − *R*^2^) (Yin and Fan, 2001). The resulting Δ*R*^2^ is an approximately unbiased estimate of the *increase* of the proportion of shared variance relative to the situation characterized by the null hypothesis. We also use the transformed set correlation log(1 − *R*^2^) for the purpose of averaging results across different stimuli, as well as across different speakers (for *R*^2^_SL*i*_).

The permutation approach is furthermore useful because it provides a straightforward and precise way to correct for multiple testing, for example for the speaker–listener analysis at different time lags. Since the same set of permutations is used to assess the null distributions of all test statistics, a corrected significance threshold can be determined by using the permutation distribution of the maximum effect across all lags.
